# Long-term mortality and risk factors for development of end-stage renal disease in critically ill patients with and without chronic kidney disease

**DOI:** 10.1186/s13054-015-1101-8

**Published:** 2015-11-03

**Authors:** Claire Rimes-Stigare, Paolo Frumento, Matteo Bottai, Johan Mårtensson, Claes-Roland Martling, Max Bell

**Affiliations:** Section of Anaesthesia and Intensive Care Medicine, Department of Physiology and Pharmacology, Karolinska Institute, Stockholm, Sweden; Department of Anaesthesia, Surgical Services and Intensive Care (ANOPIVA) F2, Karolinska University Hospital, Solna, 171 76 Stockholm Sweden; Unit of Biostatistics, Institute of Environmental Medicine (IMM), and Karolinska Institute, Stockholm, Sweden; Department of Intensive Care, Austin Hospital, Melbourne, VIC Australia

## Abstract

**Introduction:**

Prevalence of chronic kidney disease (CKD) amongst intensive care unit (ICU) admissions is rising. How mortality and risk of end-stage renal disease (ESRD) differs between those with and without CKD and with acute kidney injury (AKI) is unclear. Determining factors that increase the risk of ESRD is essential to optimise treatment, identify patients requiring nephrological surveillance and for quantification of dialysis provision.

**Method:**

This cohort study used the Swedish intensive care register 2005–2011 consisting of 130,134 adult patients. Incomplete cases were excluded (26,771). Patients were classified (using diagnostic and intervention codes as well as admission creatinine values) into the following groups: ESRD, CKD, AKI, acute-on-chronic disease (AoC) or no renal dysfunction (control). Primary outcome was all-cause mortality. Secondary outcome was ESRD incidence.

**Results:**

Of 103,363 patients 4,192 had pre-existing CKD; 1389 had ESRD; 5273 developed AKI and 998 CKD patients developed AoC. One-year mortality was greatest in AoC patients (54 %) followed by AKI (48.7 %), CKD (47.6 %) and ESRD (40.3 %) (*P* < 0.001). Five-year mortality was highest for the CKD and AoC groups (71.3 % and 68.2 %, respectively) followed by AKI (61.8 %) and ESRD (62.9 %) (*P* < 0.001). ESRD incidence was greatest in the AoC and CKD groups (adjusted incidence rate ratio (IRR) 259 (95 % confidence interval (CI) 156.9–429.1) and 96.4, (95 % CI 59.7–155.6) respectively) and elevated in AKI patients compared with controls (adjusted IRR 24 (95 % CI 3.9–42.0); *P* < 0.001). Risk factors independently associated with ESRD in 1-year survivors were, according to relative risk ratio, AoC (356; 95 % CI 69.9–1811), CKD (267; 95 % CI 55.1–1280), AKI (30; 95 % CI 5.98–154) and presence of elevated admission serum potassium (4.6; 95 % CI 1.30–16.40) (*P* < 0.001).

**Conclusions:**

Pre-ICU renal disease significantly increases risk of death compared with controls. Subjects with AoC disease had extreme risk of developing ESRD. All patients with CKD who survive critical care should receive a nephrology referral.

**Trial registration:**

Clinical trials registration number: NCT02424747 April 20th 2015.

**Electronic supplementary material:**

The online version of this article (doi:10.1186/s13054-015-1101-8) contains supplementary material, which is available to authorized users.

## Introduction

As the demographic of the intensive care unit (ICU) population changes, more patients present with pre-existing renal dysfunction [1, 2]. Chronic kidney disease (CKD) is defined by the Kidney Disease Improving Global Outcomes (KDIGO) as an abnormality of kidney function or structure present for more than 3 months, and is classified according to glomerular filtration rate (GFR) and proteinuria to stages 1–5. Patients in stage 5 who have a loss of renal function (GFR <15 ml/min per 1.73 m^2^) requiring dialysis are referred to as having end-stage renal disease (ESRD) [3]. CKD and ESRD are associated with increased risk of hospitalisation, cardiovascular disease and death compared with individuals without renal dysfunction, and these diagnoses may affect outcomes following ICU admission [4–7]. How the risk of death for patients with CKD and ESRD differs from those with de novo acute kidney injury (AKI), and what impact acute-on-chronic disease (AoC) may have, has not been fully investigated in ICU populations. A number of studies have addressed mortality in hospitalised and community-based populations with renal dysfunction; however, outcomes may not be generalisable to the ICU where the panorama of diseases and illness severity precipitating admission differ [8, 9]. Studies of ICU patients often lack comparison ICU cohorts and few have described long-term follow-up. Evidence suggests that illness severity scoring systems may overestimate mortality risk in ICU patients with pre-existing renal impairment [2, 10]. This may lead to overly negative prognostication and restrictive treatment. Emerging evidence suggests that ICU outcomes for patients with ESRD may be better than previously assumed and superior to survival in patients with AKI [11, 12].

How factors related to ICU admission are associated with ESRD risk has not been fully elucidated. In a large Danish cohort, AoC was found to increase the risk of ESRD compared with CKD per se; surprisingly, de novo AKI was found to carry a greater cumulative risk of ESRD than isolated CKD [13]. Identification of pre-ICU risk factors, such as premorbid CKD and other co-morbidities, combined with intra-ICU risk factors such as AoC and de novo AKI is essential to improve treatment strategies during critical care and to identify patients who merit continuing nephrological surveillance. This is vital because, unfortunately, post-ICU nephrological follow-up of patients with pre-existing renal dysfunction or de novo AKI is not currently routine practice in Sweden or in many other countries.

The Swedish Intensive care register (SIR) database has near complete coverage of all Swedish ICU admissions. The use of SIR and other national registers allowed reliable identification both of subjects with pre-ICU renal dysfunction and uniquely a control population with no recorded renal disease prior to, or whilst in, ICU. We previously used this cohort to examine risk of death and post-ICU renal impairment in patients without premorbid renal disease suffering from AKI [14]. In the present study we aimed to determine the long-term risk of death and ESRD in ICU patients with and without pre-existing renal dysfunction and to compare their risks to patients with AKI and with those who develop AoC disease. Additionally, we aimed to identify premorbid and ICU admission parameters predicting development of ESRD in order to pinpoint patients requiring nephrological follow-up at discharge.

## Method

### Study design

We used prospectively collected data from SIR and other Swedish national health registries. The Stockholm regional ethics committee granted ethical approval and informed consent was deemed unnecessary due to the scale and observational nature of the study. The study was performed in accordance with the ethical standards laid down in the 1964 Declaration of Helsinki and its later amendments.

### Study cohort

We conducted an observational study from January 2005 to January 2010, using data from SIR. We included all first ICU admissions of adult patients (>18 years). We excluded patients with missing disease severity scores, intervention codes and/or diagnosis codes for AKI from International Classification of Diseases version 10 (ICD-10).

We used the unique 10-digit Swedish identification number to cross-link SIR data with the following national registers, previously described in detail [14] and in (Additional file 1):

1. The Swedish cause of death register to obtain details of all-cause mortality.
2. The national patient register (NPR) was utilised to obtain the subjects comorbidities using ICD-10 codes which we then classified according to the Charlson comorbidity index [15].
3. The Swedish renal register (SRR) provided data on individuals with ESRD prior to and post-ICU admission.

Primary outcome was mortality up to 5 years. Secondary endpoint was ESRD.

### Definitions

We classified patients based on their pre-ICU renal status as having no renal disease, CKD or ESRD. Premorbid creatinine levels or GFR estimates were not available Patients were identified as having CKD if ICD-10 codes for moderate to severe renal disease according to the Charlson criteria (detailed in Additional file 2) were present in NPR. Current SRR guidelines recommend that only patients with CKD grade 3b or higher should be registered in NPR; these cases are patients with severe CKD. ESRD was recorded if subjects were registered in the SRR. Subjects were further grouped according to the presence or absence of AKI during ICU admission. Patients with no prior renal dysfunction who fulfilled any of the criteria below were recorded as having de novo AKI and are referred to hereafter as AKI. Subjects with premorbid CKD additionally meeting criteria 1, 2 or 3 below were considered to have AoC disease. Patients with no recorded renal disease are referred to as the control group, whilst patients in the CKD, ESRD and AKI groups are collectively described as having renal dysfunction.

Criteria for AKI:

1. Intermittent haemodialysis (IHD) or continuous renal replacement therapy (CRRT) reported in SIR.
2. The diagnosis “acute renal failure” recorded within the Acute Physiology and Chronic Health Evaluation (APACHE) II score, defined as a creatinine increase by >1.5 times from baseline (known to the diagnosing doctor) with urine output <410 ml in 24 hours.
3. Diagnosis code “acute kidney failure” N17 in ICD-10 assigned at discharge.
4. A serum creatinine >354 μmol/l (KDIGO grade 3) recorded on admission in APACHE II, Simplified Acute Physiology Score (SAPS)-II or SAPS-III scoring systems.

Patients were considered to have developed ESRD if they were registered in the SRR 3 months or more after admission to ICU.

Data from the Swedish cause of death register was available until 31 December 2011 and maximum follow-up for primary outcome was 7 years. Data from other national registers were available until 31 December 2010 and therefore the maximum follow-up for secondary analysis was 6 years.

### Statistical analysis

We report continuous data as medians with interquartile range (IQR). Categorical data are expressed as counts and percentages. The Mann–Whitney test was used to compare distributions of continuous variables at baseline between each group and the no renal disease (control) cohort. The Fisher’s exact test was used to compare prevalence of comorbidities between groups. A two-sided *P* value <0.05 was considered significant.

### Primary analysis

We considered time from ICU admission to death or end of follow-up (31 December 2011 for death or 31 December 2010 for secondary analysis), whichever occurred first. Information regarding emigration was unavailable. Survival curves were estimated by the Kaplan-Meier method and the log-rank test was used to verify equality of survivor functions between subgroups. We tested for proportionality of survival curves using Schoenfeld residuals and found evidence of non-proportionality; proportional hazard regression was therefore inappropriate and we instead used Poisson regression, which more easily allowed modelling time varying covariates and non-proportional hazards. We present incidence rate ratios (IRR).

### Multivariable analysis

Potential confounders were considered on the basis of prior knowledge of AKI and CKD and on whether inclusion of the covariates to the models changed estimates of log relative risk by >10 % [16]. We selected and tested age, sex, SAPS-III score (the scoring system most often recorded), acute surgery and the Charlson comorbidity groups as potential confounders and adjusted for these in our sensitivity analysis of subgroups. We present two models of multivariable analyses for primary outcome: a fully adjusted model which includes SAPS-III score (Model 2) and a partially adjusted model (Model 1), which excluded SAPS-III score. Some covariates only significantly changed log relative risk in the fully adjusted model in the presence of SAPS III and are therefore not present in model 1.

### Survival percentiles

Laplace regression was used to estimate the number of days of survival to event (death or ESRD) for the fifth, tenth, twentieth and thirtieth centiles in all groups [17].

### Secondary analysis

Secondary analyses were performed in a similar manner to the primary analysis. Time from admission to ESRD was considered, with censoring occurring at the point of death or end of follow-up, whichever occurred first. A multivariable analysis model is presented for secondary outcome.

Additionally, a polynomial logistic regression was performed to identify predictors of development of ESRD at 1 year in 1-year survivors. The model included no censored data. All patients were followed up for at least 1 year; that is, no patient was censored before the end of the first year. This competing risks model included four-level polytomous outcomes defined as death, ESRD, ESRD and death or no negative outcome, with the latter being the reference outcome. Stepwise backwards elimination was used to construct the model at the significance level of *P* > 0.1. Covariates were selected on the basis of a priori knowledge of AKI and CKD and covariates which changed estimates of log relative risk by >10 %, including available laboratory data and comorbidities and demographic data.

Relative risk ratios (RRR) are reported because multiple outcomes were possible. RRR are the ratio of relative risks for the outcome ESRD versus base category (survival without ESRD) for each given covariate pattern compared with a reference category. This reference category was male, with no comorbidities (according to Charlson index), normal admission potassium (3.9–4.59 mmol/l) and no renal disease (pre- or peri-ICU).

The polytomous (competing risk) model enabled prediction of the probability of the outcome ESRD occurring and allowed creation of a binary variable ESRD/no ESRD. The sensitivity and specificity of this prediction was investigated with respect to this binary outcome by using the receiver operating characteristic (ROC) curve. The area under the ROC curve (AUC) was used to assess discrimination.

Analysis was performed using Stata version 12 (StataCorp LP, College Station, TX, USA).

## Results

**Fig. 1:**
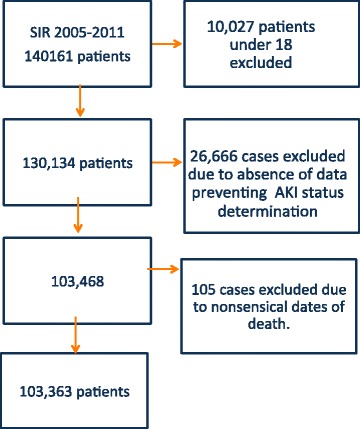
Flow chart of SIR cohort and excluded cases. *AKI* Acute kidney injury, *SIR* Swedish Intensive care register

We identified 130,134 first admissions between 2005 and 2011. A flow chart detailing case exclusion is shown in Fig. 1. A total of 103,363 patients were included in the final analyses. Baseline characteristics and outcome for patients excluded due to insufficient data are presented in Additional file 3.

Overall, 4,192 (4.1 %) patients had pre-morbid CKD. Of these, 998 (23.8 %) developed AoC renal disease. In total, 1389 of 103,363 (1.34 %) patients were identified as having ESRD prior to admission; 5273 subjects developed (severe) de novo AKI, whilst the remaining patients (92,509) were considered to have had no renal disease. Characteristics of these patients are presented in Table 1.

**Table 1:** Baseline characteristics of the cohort according to renal disease status

Baseline characteristics	All	No renal disease	AKI	*P* value	ESRD	*P* value	Chronic only	*P* value	Acute-on-chronic	*P* value
	n = 10,363	n = 92,509	n = 5273		n = 1389		n = 3194		n = 998	
Demographics										
Age in years, mean (SD)	60.2 (19.3)	59.3 (19.6)	67.9 (14.2)	<0.001	61.1 (14.3)	<0.001	71.1 (13.6)	<0.001	70.4 (11.4)	<0.001
Age in years, median (IQR)	64 (47–76)	63 (45–75)	70 (61–78)		63 (52–72)		63 (52–72)		72 (64–81)	
Length of ICU stay (hours), median (IQR)	24 (13–60)	23 (12–53)	68 (26–189)	<0.001	26 (15–58)	<0.001	27 (15–63)	<0.001	64 (25–156)	<0.001
Women, n (%)	44,480 (43.0)	40,411 (43.6)	2086 (39.6)	<0.001	500 (36.0)	<0.001	1140 (35.7)	<0.001	343 (34.4)	<0.001
Admissions per patient, mean (SD)	1.3 (0.88)	1.31 (0.89)	1.29 (0.74)	0.19	1.36 (0.86)	<0.001	1.35 (0.88)	0.005	1.30 (0.74)	0.55
Admissions per patient, median (IQR)	1 (1–1)	1 (1–1)	1 (1–1)		1 (1–1)		1 (1–1)		1 (1–1)	
Laboratory data^a^ (median values given with IQR, n denotes number of patients in whom this data was available)										
Highest potassium (mmol/l)	4.2 (3.9–4.6)	4.1 (3.9–4.5)	4.7 (4.1–5.5)	<0.001	4.8 (4.2–5.5)	<0.001	4.4 (4.0–5.0)	<0.001	4.9 (4.3–5.7)	<0.001
n	18,743	16,069	1605		273		534		262	
Highest sodium (mmol/l)	139 (136–142)	139 (136–142)	137 (134–141)	<0.001	138 (135–140)	<0.001	139 (136–142)	0.65	138 (135–141)	<0.001
n	18,792	16,121	279		270		535		262	
Lowest sodium (mmol/l)	136 (133–139)	136 (133–139)	133 (130–137)	<0.001	134 (132–137)		136 (133–140)		134 (130–137)	
n	18,797	16,121	1604							
Highest bilirubin (μmol/l)	10 (6–18)	10 (6–17)	14 (8–26)	<0.001	10 (6–17)	0.92	10 (7–17)	0.42	12(7–22)	<0.001
n	19,797	16,863	1768		207		633		326	
Lowest arterial pH	7.36 (7.29–7.41)	7.36 (7.30–7.41)	7.29 (7.19–7.38)	<0.001	7.35 (7.2–7.4)	<0.001	7.34 (7.25–7.4)	<0.001	7.28 (7.17–7.36)	<0.001
n	47,318	40,449	3968		598		1586		717	
Lowest bicarbonate (mmol/l)	22 (19–25)	23 (20–25)	18 (14–22)	<0.001	23 (19–24)	0.213	21 (18–25)	0.002	19(14–21)	<0.001
n	4566	3662	593		62		134		115	
Maximum creatinine (μmol/l)	85 (65–125)	80 (63–105)	254 (164–422)	<0.001	460 (275–673)	<0.001	185 (129–277)	<0.001	363 (238–553)	<0.001
n	54,655	47,441	4076		660		1738		740	
Maximum urea (mmol/l)	8 (4.9–14.3)	6.7 (4.3–10.1)	17.5 (11–26)	<0.001	19.4 (15–25)	<0.001	16.6 (11.9–24.5)	<0.001	27 (19.3–36.6)	<0.001
n	3519	2717	532		62		104		104	
Lowest thrombocyte count (× 10^9^/l)	228 (166–296)	230 (170–296)	199 (123–291)	<0.001	217 (153–286)	0.003	220 (152–292)	<0.001	203 (131–277)	<0.001
n	37,438	32,586	2695		421		1217		519	
Disease severity scoring system (median values given with IQR, n denotes number of patients in whom this data was available)										
APACHE II score	15 (9–22)	14 (8–20)	25 (20–32)	<0.001	22 (17–28)	<0.001	19 (14–26)	<0.001	27 (21–33)	<0.001
n	19,962	17,231	1620		284		564		263	
SAPS-II score	27 (0–44)	25 (0–41)	55 (42–70)	<0.001	35 (16–52)	<0.001	40 (23–53)	<0.001	56 (43–68)	<0.001
n	17,074	15,376	764		269		503		162	
SAPS-III score	54 (44–65)	52 (43–63)	68 (59–77)	<0.001	60 (51–71)	<0.001	64 (55–72)	<0.001	69 (60–78)	<0.001
n	20,970	18,326	1437		195		736		276	
Interventions^b^										
Invasive mechanical ventilation, n (%)	8621 (8.3)	7155 (7.7)	994 (18.9)	<0.001	93 (6.7)	0.156	201 (6.3)	0.002	178 (17.8)	<0.001
Acute surgery, n (%)	8379 (8.1)	7282 (7.8)	662 (12.6)	<0.001	109 (7.8)	1.0	228 (7.14)	0.132	98 (9.82)	0.029
Elective surgery, n (%)	5889 (5.7)	5237 (5.7)	295 (5.6)	1.0	83 (6.0)	0.6	201 (6.3)	0.111	69 (6.9)	0.098
Comorbidities										
Charlson comorbidity score, mean^c^ (SD)	2,03 (2.42)	1.8 (2.2)	2.6 (2.5)	<0.001	5.2 (2.5)	<0.001	5.8 (2.6)	<0.001	5.6 (2.6)	<0.001
Charlson score with renal points removed, mean (SD)	1.92 (2.3)	1.78 (2.2)	2.6 (2.5)	<0.001	3.2 (2.4)	<0.001	3.8 (2.6)	<0.001	3.6 (2.6)	<0.001
Myocardial infarction, n (%)	14,605 (14.1)	11,896 (12.9)	986 (18)	<0.001	387 (28.0)	<0.001	1047 (32.8)	<0.001	289 (29.0)	<0.001
Congestive cardiac failure, n (%)	16,281 (15.7)	12,521 (13.5)	1324 (25.1)	<0.001	438 (31.5)	<0.001	1563 (48.9)	<0.001	435 (43.6)	<0.001
Peripheral vascular disease, n (%)	10,948 (10.6)	8921 (9.6)	704 (13.4)	<0.001	348 (25.0)	<0.001	761 (23.8)	<0.001	214 (21.4)	<0.001
Cerebrovascular disease, n (%)	17,742 (17.2)	15,658 (16.9)	808 (15.3)	<0.001	310 (22.3)	<0.001	789 (24.7)	<0.001	177 (17.7)	0.497
Dementia, n (%)	2070 (2.0)	1840 (2.0)	99 (1.9)	0.612	16 (1.2)	0.03	100 (3.13)	<0.001	15 (1.50)	0.359
COPD, n (%)	14,841 (14.4)	12,999 (14.1)	808 (15.3)	0.010	150 (10.8)	<0.001	696 (21.8)	<0.001	188 (18.8)	<0.001
Rheumatological disease, n (%)	4006 (3.8)	3311 (3.6)	269 (5.1)	<0.001	93 (6.7)	<0.001	262 (8.2)	<0.001	71 (7.1)	<0.001
Peptic ulcer disease, n (%)	6729 (6.5)	5693 (6.2)	423(8.0)	<0.001	154 (11.2)	<0.001	362 (11.3)	<0.001	97 (9.7)	<0.001
Cancer, n (%)	18,175 (17.6)	15,726 (17.0)	1262 (24.0)	<0.001	217 (15.7)	0.183	738 (23.1)	<0.001	232 (23.3)	<0.001
Metastatic disease, n (%)	3747 (3.6)	1918 (3.6)	304 (4.7)	<0.001	24 (1.7)	<0.001	127 (4.0)	0.246	28 (2.8)	0.199
Mild liver disease, n (%)	5272 (5.1)	4504 (4.9)	369 (7.0)	<0.001	123 (8.9)	<0.001	210 (6.6)	<0.001	66 (6.6)	0.015
Moderate or severe liver disease, n (%)	2436 (2.4)	3319 (2.1)	249 (5.8)	<0.001	31 (2.2)	0.64	125 (3.9)	<0.001	58 (5.8)	<0.001
Uncomplicated diabetes, n (%)	16,684 (16.1)	13,168 (14.2)	1372 (26.2)	<0.001	537 (38.7)	<0.001	1215 (38.0)	<0.001	392 (39.3)	<0.001
Diabetes with complications, n (%)	6756 (6.5)	4660 (5.0)	523 (9.9)	<0.001	499 (35.9)	<0.001	806 (25.2)	<0.001	268 (26.9)	<0.001
Paraplegia, n (%)	2004 (1.9)	1787 (1.9)	90 (1.7)	0.287	29 (2.07)	0.624	78 (2.44)	0.043	20 (2.00	0.817
HIV, n (%)	137 (0.13)	125 (0.14)	4 (0.08)	0.328	3 (0.22)	0.44	5 (0.16)	0.63	0 (0)	0.65

^a^Laboratory data was obtained from the severity scorings systems APACHE II, SAPS-II and SAPS-III. APACHE II and SAPS-II record the highest or lowest values during the first 24 hours of ICU admission, whilst SAPS-III records values from 1 hour before until 1 hour after ICU admission. Values for scoring systems were not available in all patients; n denotes the number of patients in which this information was recorded

^b^Intervention codes were also underreported and therefore the number of patients in which these data were available is detailed in the table. Reporting of all other baseline characteristics is complete

^c^Charlson score is not age adjusted

*P* values compared to no renal disease group

*AKI* Acute kidney injury, *APACHE* Acute Physiology and Chronic Health Evaluation, *COPD* Chronic obstructive pulmonary disease, *ESRD* End-stage renal disease, *IQR* Interquartile range, *SAPS* Simplified Applied Physiology Score, *SD* Standard deviation

The median age of the cohort was 64 years. Patients with CKD and de novo AKI were significantly older than controls (74 and 73 years versus 63 years; *P* <0.001). The median length of ICU stay (LOS) was greatest for AKI patients (68 hours) and all groups with renal dysfunction had longer LOS than the controls (23 hours; *P*< 0.001). ESRD patients were younger (63 years; *P*< 0.001) than all other renal disease groups (*P* values in Table 1 refer only  to comparison of each group to the no renal disease (control) group). Test of significance between renal dysfunction groups are not displayed. The groups with ESRD and CKD had significantly shorter lengths of stay (26 and 27 hours, respectively) compared to all other renal disease groups (*P* < 0.001). The cohort consisted of 43 % women. Men were more likely than women to have pre-existing renal dysfunction (64 % of CKD and 64.3 % of ESRD patients were male; *P*< 0.001).

Illness severity scores were significantly higher in patients with renal dysfunction versus controls and were highest in those with AoC renal disease (SAPS-III 69 versus 52; *P* < 0.001). The group with ESRD had significantly lower severity scores than all other renal dysfunction groups (*P* < 0.05).

Interventions were underreported, but AKI patients and the AoC group differed from controls by having higher rates of invasive ventilation (18.9 and 17.8 versus 7.7 %; *P* < 0.001) and emergency surgery (12.6 and 9.2 versus 7.8 %; *P* < 0.001).

Compared with controls, patients with renal dysfunction had a significantly greater number of comorbidities and higher Charlson score; CKD subjects had the highest mean adjusted score (3.8 versus 1.8; *P* < 0.001). Cardiovascular disease, myocardial infarction, congestive cardiac disease and diabetes were more common amongst those with CKD compared with other groups. Subjects with pre-existing ESRD had less congestive cardiac failure, COPD and malignant disease than other patients with renal dysfunction (Table 1).

### Primary outcome

Follow-up for primary outcome was up to 7 years, with a median time of 2.1 years, whilst for secondary outcome median follow-up was 1.3 years.

During follow-up 37,836 (36.6 %) patients died. Rates of all-cause crude mortality were highest in patients with AoC renal disease who had a mortality rate ratio (MRR) relative to the control group of 3.53 (*P* < 0.001) and this differed significantly from subjects with both de novo AKI and CKD, where MRR compared to controls were 2.87 and 2.99, respectively (*P* < 0.001) (Table 2). The risk of death for ESRD patients was elevated compared with controls (MRR 2.08) but significantly lower than for patients with both CKD and AKI (*P* < 0.001). Multivariate analysis reduced estimates of MRR although they remained significantly elevated in all renal dysfunction groups compared to controls (*P* < 0.001). Full adjustment showed MRR for ESRD to be higher than for AKI (1.46 versus 1.15; *P* < 0.001).

**Table 2:** Primary outcome; multivariable Poisson regression analysis of risk of death according to renal function status

Group	n	Deaths	Person years	Mortality rate deaths/person-year (95 % CI)	Crude MRR (95 % CI)	Adjusted MRR^a^ (95 % CI)	Adjusted MRR^b^ (95 % CI)
All	103,363	37,836	2.5 × 10^5^	0.151 (0.150–0.153)			
No renal disease	92,509	31,530	2.3 × 10^5^	0.135 (0.134–0.137)	1	1	1
AKI	5273	2943	7.6 × 10^3^	0.387 (0.374–0.402)	2.87 (2.76–2.97)	2.14 (2.06–2.22)	1.15 (1.09–1.21)
Chronic only	3194	2002	4.9 × 10^3^	0.405 (0.387–0.423)	2.99 (2.86–3.13)	1.75 (1.71–1.86)	1.26 (1.17–1.36)
Acute-on-chronic	998	619	1.3 × 10^3^	0.478 (0.442–0.518)	3.53 (3.26–3.33)	2.36 (2.18–2.56)	1.38 (1.24–1.54)
ESRD	1389	782	2.8 × 10^3^	0.281 (0.26–0.30)	2.08 (1.94–2.23)	2.13 (1.98–2.30)	1.46 (1.29–1.67)

MRR are relative to patients in the no renal disease group

^a^Model 1: adjusted for age, gender, myocardial infarction and diabetes mellitus with complications.

^b^Model 2: fully adjusted model, adjusted for age, gender, SAPS-III score, myocardial infarction, cerebrovascular disease, diabetes mellitus with complications, moderate to severe liver disease, cancer and dementia.

*AKI* Acute kidney injury, *CI* Confidence interval, *ESRD* End-stage renal disease, *MRR* Mortality rate ratio

**Fig. 2:**
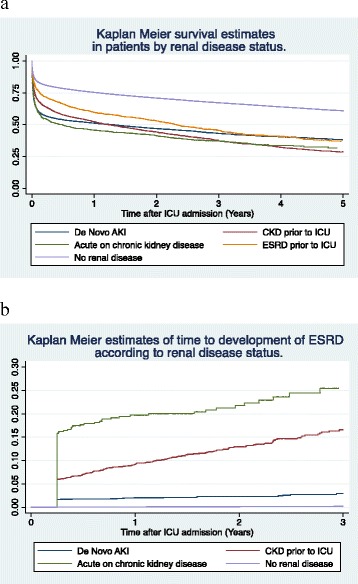
Kaplan-Meier curves showing **a** 5-year survival and **b** 3-year risk of developing ESRD according to renal disease status. Note that official registration of ESRD occurred at least 3 months after ICU admission and therefore there is a sudden steep decline in curve b corresponding to this time point. *AKI* Acute kidney injury, *CKD* Chronic kidney disease, *ESRD* End-stage renal disease, *ICU* Intensive care unit

Kaplan-Meier estimates showed 90-day mortality to be highest in the AoC and AKI groups (46.2 and 43.5 %) whilst for the ESRD group it was 29 %. However, this increased to 40.3 % by 1 year and was 62.9 % at 5 years, similar to the mortality rate for AKI (61.8 %). Patients with CKD had the highest 5-year mortality rate of 71.3 % (Table 3 and Fig. 2a).

**Table 3:** Primary outcome; Kaplan-Meier mortality estimates at specific time points according to renal function status

Group	Mortality probability (%)							
	90 days	95 % CI	1 year	95 % CI	3 years	95 % CI	5 years	95 % CI
No renal disease	19.3	19.1–19.6	24.6	24.4–24.9	29.1	28.8–29.4	39.1	38.7–39.5
AKI	43.5	42.2–44.9	48.7	47.4–50.1	53.0	51.6–54.4	61.8	60.0–64.6
Chronic only	36.8	35.1–38.5	47.7	45.9–49.4	55.7	53.9–57.4	71.3	69.1–73.4
Acute-on-chronic	46.2	43.2–49.3	54.3	51.3–57.4	58.6	55.5–61.8	68.2	64.2–72.2
ESRD	29.0	26.7–31.4	40.3	37.8–42.9	47.0	44.4–49.7	62.9	59.8–66.1

*AKI* Acute kidney injury, *CI* Confidence interval, *ESRD* End-stage renal disease

Crude survival for the fifth to thirtieth centiles analysis (Table 4) shows that 5 % of all patients died within one day of ICU admission. Twenty percent of AKI and AoC patients were dead within 5 days, whilst the first 20 % of deaths in the no renal disease group occurred by 117 days. Thirty percent of patients in the AKI, CKD and AoC groups had died by 11.0, 31.0 and 31.4 days, respectively. In contrast it took 109 days for the first 30 % of patients in the ESRD group to die.

**Table 4:** Primary outcome; crude survival centiles derived from Laplace regression according to renal function status

Group	Crude survival (days) for each given centile (95 % CI)			
	5th	10th	20th	30th
No renal disease	1.0 (0–2.0)	5.9 (4.6–7.3)	117 (108–125)	745 (725–765)
AKI	0.76 (0–2.4)	1 (0–3.6)	4 (0.8–7.0)	11 (7.5–14.5)
Chronic only	1.0 (0–4.4)	2.0 (0–7.4)	9.0 (2.5–15.5)	31.0 (23.7–38.3)
Acute-on-chronic	1 (0–4.9)	1.2 (0–7.8)	4.6 (0–12.8)	13 (3.6–22.4)
AKI on CKD	1.0	1.3	4.6	31.4
ESRD	1.0 (0–7.8)	2.8 (0–10.8)	20.3 (10.0–30.7)	109.0 (72.2–145.7)

*AKI* Acute kidney injury, *CI* Confidence interval, *CKD* Chronic kidney disease, *ESRD* End-stage renal disease

### Secondary outcome

In univariate analysis, incidence of ESRD (Table 5) was highest in the AoC group (0.138 events per person year) followed by the CKD groups (0.069 events per person year; *P* < 0.001). Crude IRR were 205 and 103, respectively. The proportion of patients with ESRD was 9.13 % in the CKD group and 19.71 % in AoC subjects at 1 year. This rose to 21.09 and 25.45 %, respectively, at 5 years. AKI patients 1-year incidence of ESRD was 2 %, increasing to 3.9 % at 5 years (Table 6; Fig. 2b).

**Table 5:** Secondary outcome; multivariable Poisson regression for risk of developing ESRD according to renal disease status

Group	Patients (n)	Events (n)	Person years	IR event/person year (95 % CI)	Crude IRR (95 % CI)	Adjusted IRR^a^ (95 % CI)
No renal disease	92,509	116	1.7 × 10 ^5^	0.0007 (0.0006–0.0008)	1	1
AKI	5273	65	5.2 × 10 ^3^	0.0125 (0.0098–0.0160)	18.6 (13.7–25.2)	24.1 (13.9–42.0)
Chronic only	3194	237	3.4 × 10 ^3^	0.069 (0.0611–0.0788)	103 (82.5–128.6)	96.4 (59.7–155.6)
Acute-on-chronic	998	111	803.1	0.1382 (0.1147–0.1665)	205.1 (158.1–266.1)	259 (156.9–429.1)

^a^Adjusted for Simplified Applied Physiology Score version 3 score, age, gender and diabetes and dementia.

*CI* Confidence interval, *IR* Incidence rate, *IRR* Incidence rate ratio

**Table 6:** Secondary outcome; Kaplan-Meier estimates of likelihood of developing ESRD at specific time points according to renal disease status

Group	Probability of ESRD (%)							
	90 days	95 % CI	1 year	95 % CI	3 years	95 % CI	5 years	95 % CI
No renal disease	0.04	0.03–0.06	0.08	0.06–0.10	0.20	0.16– 0.25	0.30	0.24–0.38
AKI	1.67	1.25–2.22	2.03	1.56–2.65	2.95	2.18–3.98	3.88	2.72–5.51
Chronic only	5.95	4.98–7.10	9.13	7.88–10.57	16.56	14.38–19.03	21.09	17.92–24.73
Acute-on-chronic	15.82	12.93–19.28	19.71	16.45–23.52	25.45	20.92–30.76	25.45	20.92–30.76

*AKI* acute kidney injury, *ESRD* End-stage renal disease, *CI* 95 % confidence interval

Multivariate analysis showed that ESRD incidence remained highest in the AoC and CKD group after adjustment IRR (259 and 96.4); for subjects with de novo AKI, risk was also elevated although to a lesser degree (adjusted IRR 24; *P* < 0.001; Table 5).

The competing-risks multinomial regression analysis showing predictors of ESRD at 1-year post-ICU admission in 1-year survivors with RRR is presented in Table 7 (predictors of other outcomes used only in order to construct the model are not presented). The RRR showed the predicted risk of developing ESRD versus survival with no ESRD. For patients with CKD it was 265.7 times higher than for those without CKD. For the AoC group it was 356.6 times higher than for patients with no renal disease.

**Table 7:** Competing risks model for predicting risk of ESRD in 1-year ICU survivors by polynomial multivariable logistic regression analysis

Covariate	Relative risk ratio^a^ (95 % CI)	*P* value
Female gender	1.12(0.48–2.63)	0.787
Congestive heart failure	0.091 (0.011–0.690)	0.020
Admission serum potassium high (>4.59)	4.6 (1.30–16.40)	0.018
AKI	30.4 (5.98–154)	<0.001
CKD	265.7 (55.1–1280)	<0.001
AoC	356.6 (69.9–1811)	<0.001

Reference category = male, no comorbidity (according to Charlson index), admission potassium (3.9–4.59), no renal disease

^a^Risk of ESRD versus survival without ESRD relative to the reference category

*AKI* Acute kidney injury, *AoC* Acute-on-chronic kidney disease, *CI* Confidence interval, *CKD* Chronic kidney disease

**Fig. 3:**
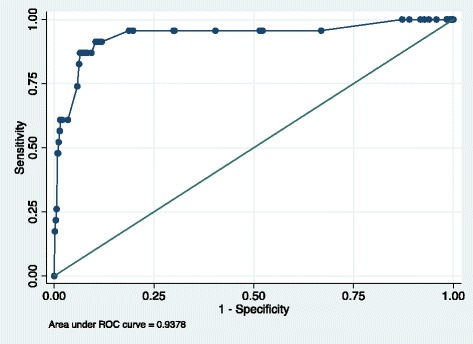
Area under the receiver operating characteristic (ROC) curve for competing-risk model predicting likelihood of ESRD among 1-year survivors

The area under the ROC curve (presented in Fig. 3) from the predicted value of the multinomial logistic regression was 0.937 (95 % CI 0.87–1.00). Significant predictors were high serum potassium on admission (RRR 4.61; 95 % CI 1.29–16.38) and presence of pre-ICU CKD )RRR 265.7; 95 % CI. 55.2–1279), AKI (30.3; 95 % CI. 5.98–154.5) or AoC (365; CI 95 % 69.9–1818). Age was not associated with likelihood of ESRD; it was modelled using cubic splines and categorised by 20-year intervals, as patients over 80 were less likely to receive chronic dialysis than younger patients. Congestive heart failure was found to be negatively associated with the risk of ESRD at 1 year; it was associated with death at 1-year (*P* < 0.001).

## Discussion

We explored long-term risks of mortality and ESRD in more than 100,000 ICU patients with or without pre-morbid CKD. Compared with patients without renal disease we found that the CKD group had a threefold crude increased risk of death, the AoC group had 3.5-fold, the ESRD group 2.1-fold and the AKI group 2.9-fold higher mortality.

In ICU survivors, subjects with CKD prior to admission had significantly elevated risk of developing ESRD in comparison to ICU controls. The highest risk was seen in the AoC group who had 259 times higher adjusted risk of developing dialysis dependence than the control group, whilst those with CKD had a 96-fold elevated risk of ESRD.

Our finding that subjects with CKD, and particularly AoC, had higher mortality rates compared with those with AKI is consistent with most other studies. A study of 9450 surgical patients found long-term survival to be significantly worse for those with AoC than for patients with AKI (hazard ratio (HR) 3.3) [8]. In a second study, Lebiedz et al. found the presence of AKI in patients with nondialysis-dependent CKD to be associated with 1-year mortality [18]. However, an observational cohort of 618 AKI patients from the PICCARD study reported better crude survival for patients with pre-existing CKD than for subjects without [19]. It was noted that these patients received earlier nephrological referral; perhaps prompt assessment led to earlier intervention.

Mortality in our study was relatively low for ESRD patients (90-day mortality, 29 %) compared with two previous studies, where 90-day mortality rates were 42 and 44.6 % [20, 21].We also found that crude mortality was lower for ESRD patients compared with AKI patients (1 year 40.3 % versus 48.7 %; *P* > 0001). However, this survival advantage was not maintained after adjustment, indicating presence of selection bias. Our ESRD population was younger, less severely ill, and had less comorbidity than other renal disease groups. Ostermann and co-workers found their ESRD population to be affected by fewer organ failures and less likely to be mechanically ventilated [22]. ESRD patients selected for admittance to ICU may represent a healthier subgroup of the ESRD population usually presenting with less severe disease than other patients. A cohort of 41,972 UK and German ICU admissions found lower mortality in ESRD than AKI patients (hospital mortality 34.5 % versus 61.6 %; *P* < 0.0001) [22]. Reviews examining this and other studies found survival to be better for ESRD patients than for AKI groups as did two subsequent original studies [11, 12, 23–25].

The most striking finding of this study was the extreme relative risk of ESRD in patients with CKD prior to ICU admission, in particular for those with AoC. This occurred despite the high competing risk of death in these groups and was confirmed by the competing risk analysis. These observations are concordant with a large cohort which found AoC patients (without renal recovery at discharge) to have a HR of 213 for developing ESRD, compared with patients with preserved kidney function [8]. Ishani et al. reported a HR of 41.2 for AoC (79.5 cases per 1000 patients) compared with controls in a cohort of over 233,000 elderly hospitalised patients [9]. Another community-based study of over 39,000 individuals found dialysis-requiring AoC increased the risk of developing ESRD by 30 % compared with CKD without AKI [26]. Clearly CKD patients have a much higher risk than the general population of developing ESRD and the risk is directly proportional to GFR reduction. Risk of developing ESRD for CKD patients has been quantified in two studies as being between 4.14 and 6.37 per 1000 person years [27, 28]. Our findings of an ESRD IR per 1000 person years of 69.0 for CKD and 138 for AoC patients admitted to ICU (presented in Table 5 as events per person year) is clearly far greater than the risk attributable to natural progression of CKD alone.

The model of covariates predicting ESRD at 1 year in survivors produced an AUC of 94 %; this simple model is more discriminatory than any currently available novel biomarkers at identifying risk of ESRD [29, 30]. Modelling of ESRD is complex due to the fact that ESRD is not purely a biological endpoint (as GFR measurement is); it requires acceptance to a treatment programme, which excludes patients on the basis of old age or comorbidity.

This study has limitations; it was affected by underreporting, a problem common to most register studies of this magnitude. AKI diagnosis, interventions and  in particular renal replacement therapy, were not always recorded, meaning that we were not exhaustively able to identify all patients with AKI and AoC disease. However, cases where these diagnoses were recorded should represent those with the most severe disease. As a result, some patients with mild acute disease may have been misclassified to no renal disease or CKD only groups. This would result in a type one error bringing differences between groups towards the null. Despite this, statistically significant differences were observed between the cohorts suggesting that identification of subjects was predominantly correct. We identified patients previously diagnosed with CKD using the NPR, but baseline creatinine and GFR measurements were unavailable, and thus some subjects may have suffered from undiagnosed CKD prior to admission to the ICU. Additionally it was not possible to acertain the exact CKD grade.

We excluded individuals with insufficient data for AKI categorisation. Analysis of the excluded subjects revealed that they were younger, with lower disease severity and had shorter LOS than the studied cohort (Additional file 3). Excluded subjects seemingly represent a healthier group, with less AKI and AoC disease; their mortality rates were significantly lower than in the study cohort.

As a matter of convention, we present the SAPS-III score in our fully adjusted regression model. However, one of the components of SAPS-III is creatinine which itself was used to define AKI and non-AKI groups. We were unable to remove “renal points” which may lead to over-adjustment. Similarly, including other covariates common to SAPS-III such as age, acute surgery and malignancy in regression models may compound risk of over-adjustment.

The strengths of this study lie in the use of well validated, reliable national databases, which allowed us to categorise subjects based on pre-ICU renal status and almost uniquely permitted identification of a large ICU control population for comparison. Completeness of outcome data enabled us to accurately describe long-term mortality and ESRD incidence. Thus, we suggest that the study has a high degree of internal validity. The scale of the cohort and high coverage of ICU admissions from both general and speciality ICUs should allow generalisation to other national ICU populations making the external validity of our study high.

## Conclusion

In one of the largest studies examining the effect of pre-ICU renal disease status on outcome after ICU, we demonstrated that ESRD patients have mortality similar to that of AKI subjects. These patients may represent a healthier subsection of the ESRD cohort. Nonetheless, overly negative prognostication for this group is not merited. Patients with prior CKD and particularly AoC were demonstrated to have elevated risk of death and a strikingly high relative risk of developing ESRD. These results establish that nephrological follow-up is imperative for all CKD patients surviving critical care and in particular those with AoC and those with elevated potassium on admission. Clearly the implications for planning and provision of nephrology and dialysis facilities are substantial because increasing numbers of CKD patients are being admitted to ICU with a greatly elevated risk of developing ESRD.

## Key messages

- Patients presenting to ICU with pre-existing renal dysfunction have a high risk of death and of developing ESRD. Patients with CKD who survive ICU should receive nephrological follow-up.

## Additional files

Additional file 1:
**Swedish health registers.** Details of the Swedish National health registers. (DOCX 30 kb) Additional file 2:
**ICD-10 codes Charlson Comorbidity renal disease.** List of ICD-10 codes used in Charlson Comorbidity Index to define (moderate to severe) renal disease. (DOCX 16 kb) Additional file 3:
**Excluded cases.** Details of excluded cases including table of demographic and outcome data. (DOCX 24 kb)

## Abbreviations

AKI: Acute kidney injury
AoC: Acute-on-chronic disease
APACHE: Acute Physiology and Chronic Health Evaluation
AUC: Area under the curve
CI: Confidence interval
CKD: Chronic kidney disease
CRRT: Continuous renal replacement therapy
ESRD: End-stage renal disease
GFR: Glomerular filtration rate
HR: Hazard ratio
ICD-10: International Classification of Diseases version 10
ICU: Intensive care unit
IHD: Intermittent haemodialysis
IQR: Interquartile range
IRR: Incidence rate ratios
KDIGO: Kidney Disease Improving Global Outcomes
LOS: Length of ICU stay
MRR: Mortality rate ratio
NPR: National patient register
ROC: rReceiver operating characteristic
RRR: Relative risk ratios
SAPS: Simplified Acute Physiology Score
SIR: Swedish Intensive care register
SRR: Swedish renal register
