# Supplementary material for: Long-term mortality and risk factors for development of end-stage renal disease in critically ill patients with and without chronic kidney disease
Source: Crit Care. 2015 Nov 3;19:383. doi: 10.1186/s13054-015-1101-8 (PMC4630837; doi:10.1186/s13054-015-1101-8)
Supplement: Additional file 1: — Swedish health registers. Details of the Swedish National health registers. (DOCX 30 kb) [file 13054_2015_1101_MOESM1_ESM.docx]

**Additional file 1**

**Details of the Swedish National health registers. Details of the Swedish National health registers.**

Swedish intensive care register (SIR).

SIR, which was established in 2001, receives data from intensive care units in district, county and tertiary referral centres including general, cardiothoracic, neurosurgical and burns injuries units. Since its establishment the number of units submitting data has increased, and by 2011 91% of all patients admitted to Swedish ICUs were included in SIR [13].

The submission of data relating to patient characteristics and administrative details of ICU admissions to SIR is mandatory and this data is therefore complete. Registration of other variables however is optional and data is sometimes absent. Examples of non-mandatory data include interventions, surgical codes, complications and disease severity scoring systems from which admission laboratory data was obtained. Pre-morbid biochemistry data was not available. Further details of SIR may be found in additional material Appendix A.

SIR uses national personal identification numbers to identify patients in the register and admissions to ICU without valid national personal identification are assigned a temporary number. SIR data is collected prospectively and transferred electronically to the register. A validation script identifies logical defects and returns these to each centre for re-evaluation prior to data being entered in the master database.

During the course of the study SIR used three disease severity scoring systems, including APACHE II (Acute physiology and Chronic health evaluation) used between 2008-2010, SAPS (Simplified Acute Physiology Score) version II (2005 -2009) and SAPS III (2008-2011). Additionally the admission reason according to APACHE recorded on ICU entry and ICD-10 diagnoses determined at discharge, are available in many but not all patients.

**The Swedish cause of death register.**

The Swedish cause of death register includes the deaths of all Swedish citizens and residents with a national identification number. This register is considered to be a very reliable source of data with over 99% of all deaths reported to it [14].

**The national patient register (NPR)**

NPR is a mandatory database, comprising both the in- and out-patient registers, records ICD-10 diagnosis codes, which are used as the internal debit system within the Swedish health care service. The in-patient register contains all hospital discharges in Sweden, and complete coverage has been achieved since 1987[29]. Out-patient register validation in 2011 indicated that 77% of all outpatient episodes were recorded, and private health care contacts accounted for the majority of omissions [30, 31].

**The Swedish renal register (SRR)**

SRR includes details of all individuals receiving treatment for ESRD. The database records the start and finish date for all patients receiving chronic haemo- or peritoneal dialysis and those who receive renal transplants[16]. There are around 3850 patients receiving dialysis in Sweden at any point in time

[17].
