# Supplementary material for: Long-term mortality and risk factors for development of end-stage renal disease in critically ill patients with and without chronic kidney disease
Source: Crit Care. 2015 Nov 3;19:383. doi: 10.1186/s13054-015-1101-8 (PMC4630837; doi:10.1186/s13054-015-1101-8)
Supplement: Additional file 2: — ICD-10 codes Charlson Comorbidity renal disease. List of ICD-10 codes used in Charlson Comorbidity Index to define (moderate to severe) renal disease. (DOCX 16 kb) [file 13054_2015_1101_MOESM2_ESM.docx]

Additional file 2.

ICD-10 codes used in Charlson Comorbidity Index to define (moderate to severe) renal disease.

Patients were classified as having had renal disease if any of the following ICD-10 codes were recorded in the national patient register: I12.0, I13.1, N03.2 to N03.7, N05.2 to N05.7, N18.x, N19.x, N25.0, Z49.0 to Z49.2, Z94.0, Z99.2
