# Supplementary material for: Long-term mortality and risk factors for development of end-stage renal disease in critically ill patients with and without chronic kidney disease
Source: Crit Care. 2015 Nov 3;19:383. doi: 10.1186/s13054-015-1101-8 (PMC4630837; doi:10.1186/s13054-015-1101-8)
Supplement: Additional file 3: — Excluded cases. Details of excluded cases including table of demographic and outcome data. (DOCX 24 kb) [file 13054_2015_1101_MOESM3_ESM.docx]

**Additional file 3**

**Excluded cases**.

- Summary of excluded cases
- Table 1. Comparison of characteristics and outcome of patients excluded due to insufficient data versus the included cohort.

**Summary of details of excluded cases.**

Analysis showed that these ineligible patients were somewhat younger and had significantly shorter lengths of ICU stay. The incidence of all Charlson comorbidity groups was significantly lower than in the remaining cohort with the exception of HIV infection The percentage of deleted records was highest in first years of the study and in 2006, 59% of data was excluded. As reporting improved the percentage of patients with incomplete records decreased rapidly and represented 0·6% of the 2010 cohort. Quality of reporting varied initially between intensive care units with 6 units failing to report sufficient information in more than 20% of their cases. One year mortality for the excluded patients was significantly lower than for the remaining cohort (23·7% versus 27·1% respectively (p<0.000) and rates of ESRD did not differ from the overall ICU population.

**Table 1. Comparison of characteristics and outcome of patients excluded due to insufficient data versus the included cohort.**

| **Baseline characteristics** | **Patients included**  **in the study.**  **N= 103,363** | **Excluded**  **Cases.**  **N = 26666** | **P** |
| --- | --- | --- | --- |
| Age (years) mean | 60·3 | 59·0 | <0.001 |
| Age (years) median | 64 | 62 |  |
| Length of ICU stay  (hours) median | 24 | 20 | <0.001 |
| Female (%) N= 44,480 | 43·0% | 43·9 | 0·008 |
| Number of admissions (mean) | 1·31 | 1·33 | <0.001 |
| Comorbidity (%) | | | |
| Myocardial infarction  N=14605 | 14·1 | 12·5 | <0.001 |
| Congestive cardiac failure  N= 16281 | 15·8 | 14·06 | <0.001 |
| Peripheral vascular disease  N= 10948 | 10·6 | 9·9 | <0.001 |
| Cerebro-vascular disease  N=17742 | 17·2 | 16·06 | <0.001 |
| Dementia  N= 2070 | 2·0 | 1·94 | 0·52 |
| COPD  N=14841 | 14·4 | 11·95 | <0.001 |
| Rheumatological disease  N=4006 | 3·9 | 3·41 | <0.001 |
| Peptic ulcer disease  N= 6729 | 6·5 | 6·08 | <0.001 |
| Cancer  N= 18175 | 17·6 | 15·1 | <0.001 |
| Metastatic disease  N= 3747 | 3·6 | 2·85 | <0.001 |
| Mild Liver disease  N=5272 | 5·1 | 4·7 | <0.001 |
| Moderate or severe lever disease  N= 2436 | 2·4 | 2·06 | <0.001 |
| Uncomplicated Diabetes  N=16664 | 16·1 | 14·32 | <0.001 |
| Diabetes with complications  N=6756 | 6·5 | 5·76 | <0.001 |
| Paraplegia  N=2004 | 1·9 | 1·53 | <0.001 |
| HIV N=137 | 0·13 | 0·15 | <0.001 |
| Renal disease | 5·4 | 4·3 | <0.001 |
| Outcome (%) | | | |
| Died 1 year (all cohort 27.06) | 27·06 | 23·7 | <0.001 |
| ESRD | 0·51 | 0·56 | 0·34 |
| 1-year mortality by admission year (%) | | | |
| 2005 | 25·9 | 24·9 | 0·2 |
| 2006 | 31·5 | 23·6 | <0.001 |
| 2007 | 28·9 | 23·7 | <0.001 |
| 2008 | 26·8 | 18·8 | <0.001 |
